# The prognostic value of CSCs biomarker CD133 in NSCLC: a meta-analysis

**DOI:** 10.18632/oncotarget.10964

**Published:** 2016-07-30

**Authors:** Engeng Chen, Zhiru Zeng, Bingjun Bai, Jing Zhu, Zhangfa Song

**Affiliations:** ^1^ Department of Colorectal Surgery, Sir Run Run Shaw Hospital, School of Medicine, Zhejiang University, Hangzhou, 310016, P.R. China; ^2^ Key Laboratory of Biotherapy of Zhejiang Province, 310016, P.R. China; ^3^ The Second Affiliated Hospital, School of Medicine, Zhejiang University, Hangzhou, 310009, P.R. China

**Keywords:** CD133, CSCs, NSCLC, EGFR, meta-analysis

## Abstract

The prognostic value of cancer stem cells (CSCs) marker CD133 in non-small-cell lung cancer (NSCLC) remains controversial. We performed this meta-analysis of 32 eligible studies to clarify the prognostic value of CD133 and provide evidence for CSCs hypothesis. We calculated pooled hazard ratio (HR) for survival outcomes and pooled odds ratio (OR) for clinical parameters associated with CD133 in total 3595 NSCLC patients by STATA. Our results showed that NSCLC patients with higher CD133 expression had shorter overall survival time only in Asian patients (HR = 3.80, 95% CI: 3.12–4.04, *p* < 0.001; I^2^ = 32%) but not in Caucasian patients (HR = 1.15, 95% CI: 0.88–1.52, *p* = 0.307; I^2^ = 0%), suggesting that differential prognostic value of CD133 in distinct ethnic group. We speculated that the intrinsic EGFR gene status of CSCs might be responsible for this racial difference. Additionally, we found that higher expression of CD133 was associated with poor differentiation (OR = 2.03, 95% CI: 1.32–3.14, *p* = 0.001) and lymph node metastasis (OR = 2.39, 95% CI: 1.62–3.52, *p* < 0.001) but there was no significant difference of CD133 expression between adenocarcinoma and squamous carcinoma (OR = 1.13, 95% CI: 0.93–1.38, *p* = 0.3) in NSCLC patients. These results may provide a new therapeutic perspective on the treatment of NSCLC patients according to the expression of CD133 in distinct ethnic group.

## INTRODUCTION

Incontrovertibly and unfortunately, lung cancer is the most frequent reason of cancer-related deaths all over the world [1]. It is roughly estimated that there are 1.83 million new lung cancer cases and 1.59 million deaths annually around the world [2]. Approximately 83% of lung cancer patients are non-small cell lung cancer (NSCLC) patients, which 21% of those are alive at five years [3]. More powerful methods of diagnosis and treatment are indispensable to need for lung cancer patients.

Cancer stem cells (CSCs) could divide to produce heterogeneous lineages of cancer cells and new stem cells [4], which are making up a minority portion of the solid tumors, resisting to chemotherapy and radiation, correlating with targeted drug resistance and organ metastasis [5, 6]. This notion that tumors are maintained by their own stem cells has brought about novel directions to reveal the mechanisms of occurrence, progression, drug resistance, and metastasis of tumors and further seek for effective treatments of tumors. CD133 antigen, also known as prominin-1, is a member of pentaspan transmembrane glycoproteins specifically locating to cellular protrusions [7, 8]. It has been used extensively as a biomarker of CSCs in different types of cancers, such as hepatic cancer, gallbladder cancer, breast cancer, gastric cancer, pancreatic cancer and lung cancer [9–14].

Racial difference strongly affects the molecular characteristics of lung cancer [15]. Epidermal growth factor receptor mutations (mEGFR) and kirsten rat sarcoma viral oncogene mutations (mKRAS) are the most common mutations in lung cancer [16]. Alternatively, mEGFR and mKRAS usually do not occur in the same individual and have a significant association with race. For instance, Asian population have more frequently mEGFR but Caucasian population have more frequently mKRAS [17, 18]. Furthermore, it has been demonstrated that CD133 overexpressed in gefitinib-resistant tumors (GRTs) of EGFR-mutant NSCLC [19]. Therefore, we speculate that the prognostic value of CD133 in NSCLC patients might depend on given race because of various molecular characteristics.

Previous several studies about the prognostic value of CD133 in NSCLC patients suggested that NSCLC patients with higher CD133 expression have shorter overall survival (OS) time [9, 20–26] and disease free survival (DFS) [27, 28] time. On the contrary, several studies indicated that the expression level of CD133 was no association with OS and DFS [20, 29–35]. Additional, the relationship between CD133 and clinicopathological features was also in dispute [9, 20–22, 24–27, 29, 31, 33, 35–48], such as age, gender, smoking history, T stage, lymph node metastasis, distant metastasis, TNM stage, differentiation grade, and histological type. Wu. H et al. [49] and Wang. W et al. [50] have performed a meta-analysis on the prognostic value of CD133 expression in NSCLC patients, respectively. However, only 23 studies and 13 studies were included in their meta-analysis published in 2014, respectively. Additionally, several reduplicative articles (Okudela. K [28] and Woo. T [51]; Wei. YP [24] and Zhang. HZ [52]) in their meta-analysis which may limit the reliability of conclusion. Furthermore, their studies did not clarify the source of significant heterogeneity with sufficient subgroup analysis and sensitive analysis.

We performed this meta-analysis comprehensively to obtain further evidence that the biomarker of CSCs CD133 expression level may be associated with the prognosis of NSCLC patients and try to demonstrate our speculation that the prognostic value of CD133 in NSCLC patients might depend on given race for various molecular characteristics. Further, it may provide supportive evidence for the association between the cancer stem cells and the drive gene mutations of lung cancer in clinical trials and broaden new therapeutic strategy of NSCLC.

## RESULTS

### Eligible studies

**Figure 1:**
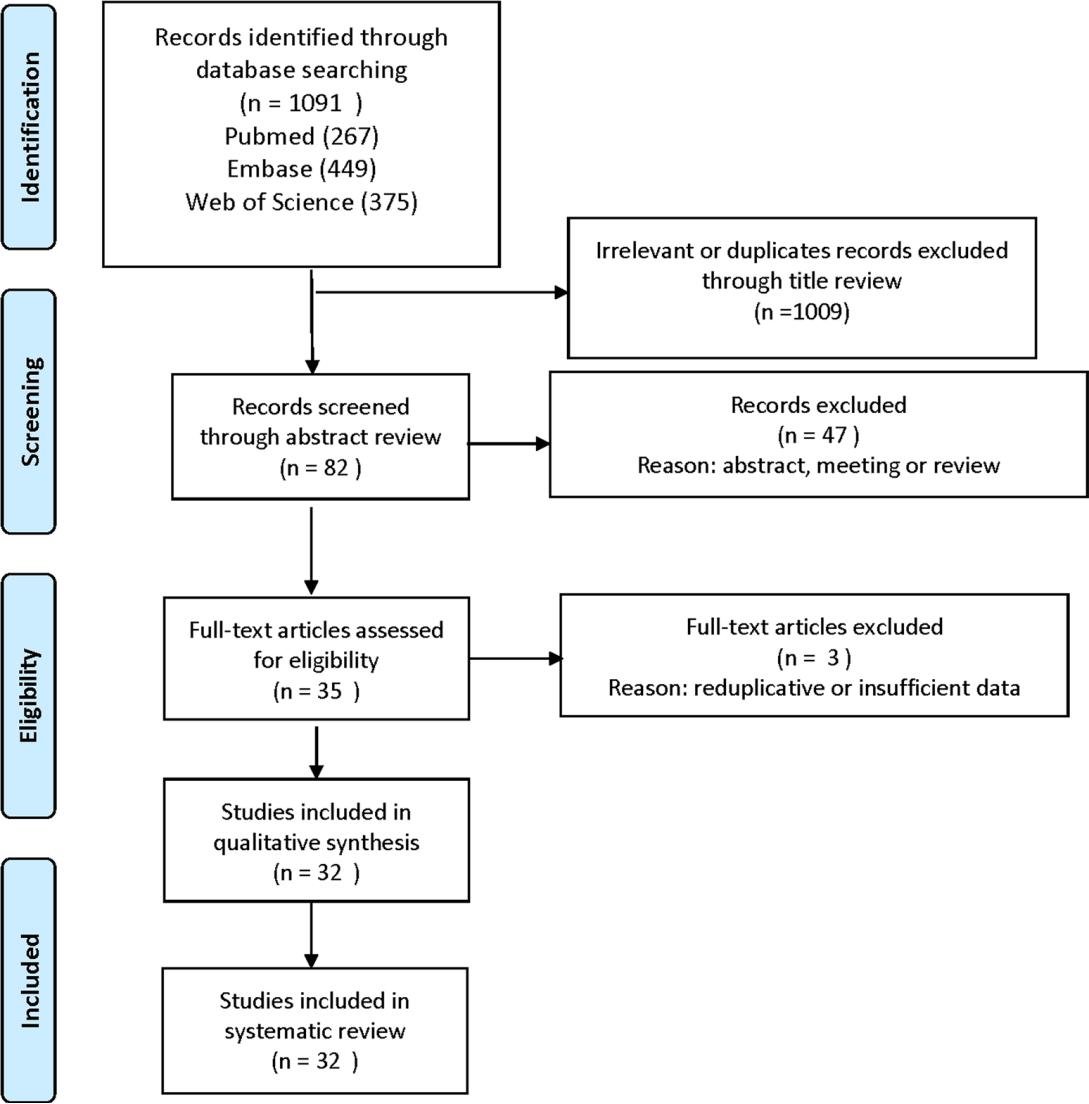
Flow diagram of study selection

We used the PRISMA 2009 flow diagram to screen the literature in Figure 1 [53]. A total of 1091 literature was identified through original searching from PubMed, Embase, and Web of Science. In total, 1009 Irrelevant and duplicate records were excluded through title review by two author independently (Engeng Chen and Zhiru Zeng). After that, we sorted the left literature through abstract review with double check and excluded 47 literature of meeting reports and reviews. Then we assessed the full text in the left thirty-five articles, and abandoned three articles that the sample data were reduplicate or insufficient. At last, 32 studies with 3595 participants were eligible in this meta-analysis.

### Study characteristics and quality assessment

The main characteristics of eligible studies were summarized in Table 1. The publication year was from 2008 to 2015. The race of study population was determined by its country. 18 Chinese studies and 4 Japanese studies composed East Asian ethnicity, and the Caucasian were from 3 Italian studies, 3 German studies, 1 Swiss study, 1 Czech study, 1 Australian study, 1 American study. Approximately 2412 male and 1183 female composed 3595 NSCLC patients in this meta-analysis, with the mean/median age range from 59 to 74.2. We defined overall survival (OS) and disease free survival (DFS) as primary endpoints. 15 studies [9, 20–26, 30–32, 42, 44, 54, 55] and 10 studies [20, 27–35] contained OS and DFS, respectively. Most of studies (29/32) used immunological histological chemistry (IHC) as experimental method for detecting CD133, and the left studies (3/32) chose quantitative real-time polymerase chain reaction (Q-PCR).

**Table 1:** Characteristics of eligible studies in the meta-analysis

Study	Year	Race	Patient(M/F)	Age	TNM	CD133 positive threshold	CD133 positive ratio	Method	Primary endpoint	NOS score
**Alamgeer.M**	2013	Caucasian	205 (125/80)	70 (median)	I	≥ 5%	68.7%	IHC	OS + DFS	8
**Bertolini.G**	2009	Caucasian	42 (29/13)	NA	I–IV	≥ 5%	23.8%	IHC/FACS	DFS	7
**Cheng.J.R**	2010	Asian	65 (58/7)	62.5 (mean)	I–III	> 10%	69.2%	IHC	NA	5
**Cortes-Dericks.L**	2012	Caucasian	64 (34/30)	62 (median)	I–III	NA	NA	qRT-PCR	DFS	8
**Gao.Y**	2015	Asian	62 (40/22)	64 (mean)	NA	≥ 5%	51.6%	IHC	NA	5
**Gottschling.S**	2013	Caucasian	100 (75/25)	63.4 (mean)	I–II	≥ 10%	18%	IHC	OS + DFS	8
**Gu.Y.P**	2010	Asian	44 (27/17)	62.5 (mean)	I–III	> 10%	68.2%	IHC	NA	5
**Herpel.E**	2011	Caucasian	86 (61/25)	64 (mean)	I–II	> 0	15.1%	IHC	OS + DFS	8
**Huang.M.J**	2015	Asian	239 (180/59)	63 (median)	I–IV	> 10%	52.3%	IHC	OS	5
**Janikova.M**	2010	Asian	121 (95/26)	NA	NA	> 10%	19%	TMA/IHC	OS + DFS	7
**Le.H.B**	2013	Asian	30 (23/7)	61.5 (median)	I–IV	NA	NA	qRT-PCR	OS	7
**Li.F**	2011	Asian	145 (111/34)	59.6 (mean)	I	> 1%	31.7%	IHC	DFS	8
**Li.H**	2011	Asian	90 (71/19)	59.5(median)	I–IV	≥ 10%	48.9%	IHC	NA	5
**Li.L.D**	2013	Asian	112 (94/18)	59.2 (median)	I–IV	NA	NA	qRT-PCR	NA	5
**Lin.X.Y**	2009	Asian	54	NA	NA	> 0	50%	IHC	NA	4
**Mizugaki.H**	2013	Asian	161 (109/52)	NA	I–IV	NA	77%	IHC	OS	7
**Okudela.K**	2012	Asian	177 (89/88)	68 (median)	I	≥ 17.5%	45.8%	IHC	DFS	8
**Pirozzi.G**	2013	Caucasian	45 (31/14)	74.2 (median)	I–III	≥ 10%	26.7%	FC/IHC/PCR	DFS	8
**Qiu.Z.X**	2015	Asian	175 (130/45)	NA	I–IV	> 3.5 score	56.6%	IHC	OS	7
**Salnikov.A.V**	2010	Caucasian	88 (79/9)	59.1 (mean)	I–III	≥ 20%	63%	IHC	OS	8
**Shien.K**	2012	Asian	30 (21/9)	NA	III	> 1%	30%	IHC	DFS	7
**Song.S.M**	2014	Asian	90 (52/38)	NA	I–III	> 4 score	61.11%	IHC	NA	5
**Sowa**	2015	Asian	239 (123/116)	67 (mean)	I–III	> 2 score	10.9%	TMA/IHC	OS	8
**Su.C.X**	2015	Asian	159 (87/72)	61 (median)	I–III	> 100 score	44%	IHC	OS	8
**Sullivan.J.P**	2010	Caucasian	207	NA	I	NA	27%	TMA/IHC	OS	7
**Sun.H.Y**	2012	Asian	67 (53/14)	60.3 (mean)	I–III	> 3 score	62. 69%	IHC	NA	5
**Tirino.V**	2009	Caucasian	89 (59/30)	NA	I–IV	NA	71.9%	IHC	NA	5
**Wang.S.G**	2012	Asian	83 (45/38)	NA	NA	NA	81.9%	IHC	NA	4
**Wei.Y.P**	2008	Asian	77 (57/20)	63 (median)	NA	> 10%	51.9%	IHC	OS	7
**Wu.S.W**	2012	Asian	305 (233/72)	59.8 (media)	I–III	> 10%	48.9%	IHC	OS	8
**Xu.Y.H**	2010	Asian	102 (66/36)	60.51 (mean)	I–IV	≥ 10%	50%	IHC	OS	8
**Yao.J**	2010	Asian	42 (24/18)	59 (median)	NA	> 10%	73.8%	IHC	NA	5

The quality of studies were assessed by Newcastle-Ottawa Quality Assessment Scale (NOS) [56]. 62.5% (20/32) of studies were more than 6 score which were deemed as high quality studies (see Supplementary Table S1 in Supplementary Material).

### Association between CD133 and OS

**Figure 2:**
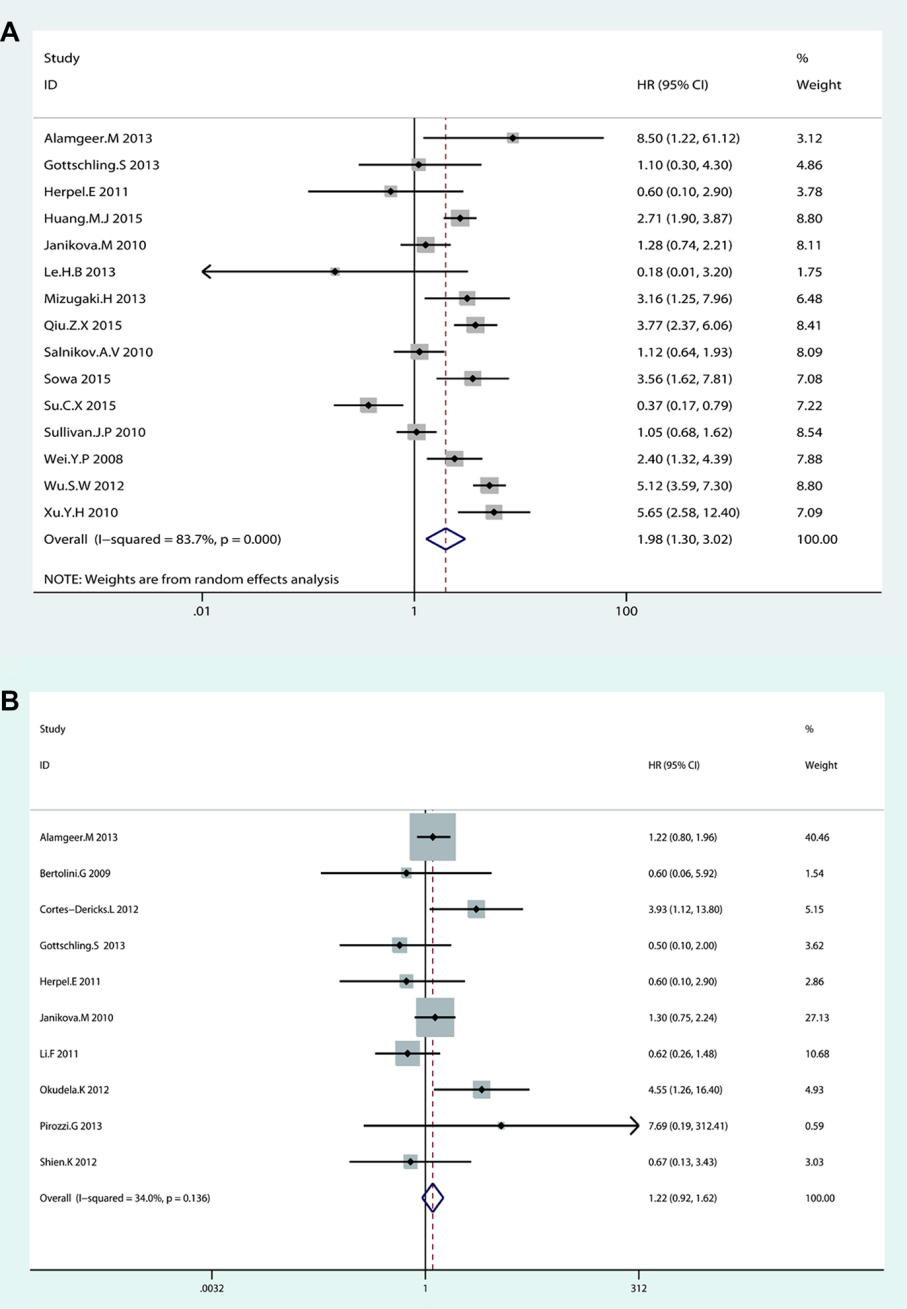
Forest plot of HRs for the association of CD133 expression in NSCLC patients with (A) OS and (B) DFS

**Figure 3:**
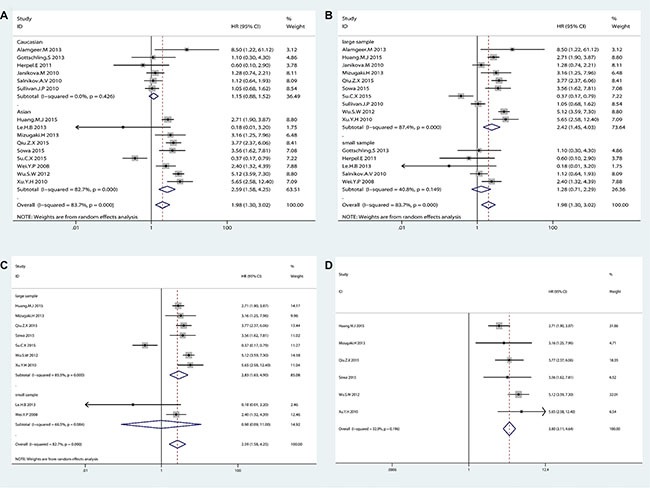
The subgroup analysis exploring the significant heterogeneity of CD133 expression with (A) OS by racial classification in NSCLC patients (B) OS by sample size classification in NSCLC patients (C) OS by sample size classification in Asian patients (D) OS after removed one study in Asian patients with large sample size

Random-effects model was used to analyze the HRs of OS from 15 eligible studies because of significant heterogeneity (I^2^ = 83.7%, *p* < 0.001). NSCLC patients with higher CD133 expression showed a shorter OS time (HR = 1.98, 95% CI: 1.30–3.02, *p* = 0.002; I^2^ = 83.7%) (Figure 2A). Subgroup analysis indicated that both race and sample size were contributed to substantial heterogeneity. The subgroup of Caucasian from 6 studies was contributed to tiny heterogeneity (I^2^ = 0%, *p* = 0.426; HR = 1.15, 95% CI: 0.88–1.52, *p* = 0.307), while the subgroup of Asian from 9 studies was contributed to subtotal heterogeneity (I^2^ = 82.7%, *p* < 0.001; HR = 2.59, 95% CI: 1.58–4.25, *p* < 0.001). (Figure 3A). In consideration of significant heterogeneity in the subgroup of Asian, we continued to divide the 9 Asian studies into groups by sample size. The pooled HR of studies with large sample size (*n* > 100) was 2.83 (95% CI: 1.63–4.90, *p* < 0.001; I^2^ = 85.5%, *p* < 0.001) (Figure 3C). Sensitive analysis in Asian studies with large sample size showed that whatever study was removed, the result was stable as before (see Supplementary Figure S1 in Supplementary Material). Furthermore, the heterogeneity decreased (I^2^ = 32%, *p* = 0.196) after dropped out one study (Su. C.X 2015) (HR = 3.80, 95% CI: 3.12–4.04, *p* < 0.001) (Figure 3D). These results suggested that NSCLC patients with higher CD133 expression had poor prognosis only in Asian patients but not in Caucasian patients, which was quite different from the conclusion of Wang. W et al. [50]. Additionally, the subgroup analysis on OS by sample size showed studies with large sample size (*n* > 100) were associated with OS (HR = 2.45, 95% CI: 1.45–4.03, *p* = 0.001; I^2^ = 87.4%, *p* < 0.001) but not studies with small sample size (≤ 100) (HR = 1.28, 95% CI: 0.71–2.29, *p* = 0.415; I^2^ = 40.8%, *p* = 0.149) (Figure 3B), and neither Wu. H et al. [24] nor Wang. W et al. [25] analyzed this in their studies.

### Association between CD133 and DFS

Fixed-effects model was used to analyze the HRs of DFS from 10 eligible studies for tiny heterogeneity (I^2^ = 34%, *p* = 0.136). No significant association was found between CD133 expression level and DFS in NSCLC patients (HR = 1.22, 95% CI: 0.92–1.62, *p* = 0.173) (Figure 2B). Though the heterogeneity was no significant (I^2^ = 34%, *p* = 0.136), the subgroup analysis by race and sample size were still performed. The results showed that there was no significant association between CD133 expression level and DFS in NSCLC patients by dividing race and sample into groups (see Supplementary Figure S2 in Supplementary Material).

### Association between CD133 and clinicopathological features

**Figure 4:**
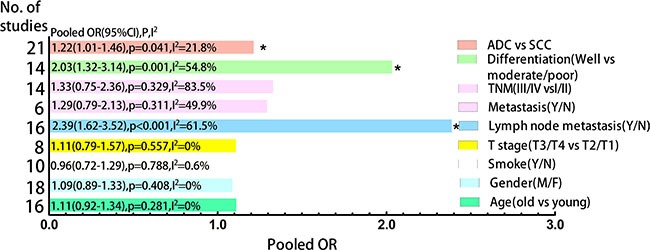
Clustered bars of pooled ORs for the association of CD133 expression with clinicopathological features in NSCLC patients

The frequency distributions of clinicopathological features in NSCLC patients with negative and positive expression of CD133 were summarized in Table 2. The pooled ORs of CD133 expression level and clinicopathological features were summarized in Figure 4. There were no associations between CD133 expression level and age, gender, smoking history, T stage, distant metastasis or TNM stage (detailed forest plot figures see Supplementary Figure S3–S8 in Supplementary Material). However, higher CD133 expression level was associated with poor/moderate differentiation (OR = 2.03, 95% CI: 1.32–3.14, *p* = 0.001; I^2^ = 54.8%, *p* = 0.007), lymph node metastasis (OR = 2.39, 95% CI: 1.62–3.52, *p* < 0.001; I^2^ = 61.5%, *p* = 0.001) and histological type (OR = 1.21, 95% CI: 1.01–1.46, *p* = 0.041; I^2^ = 21.8%, *p* = 0.18) (detailed forest plot figures see Supplementary Figure S9–S11 in Supplementary Material).

**Table 2:** The frequency distribution of clinicopathological features in NSCLC patients with negative and positive expression of CD133

Study	Age(old/young)					Gender(M/F)					Smoke(Y/N)					T stage(T3/4 vs.T1/2)					Lymph node Met (Y/N)				
**CD133**	−		+		*p*	−		+		*p*	−		+		*p*	−		+		*p*	−		+		*p*
**Alamgeer.M**	52	46	52	55	0.58	39	41	65	60	0.67	12	11	92	90	1	-	-	-	-	-	88	80	16	15	1
**Bertolini.G**	16	3	16	7	0.31	4	9	6	23	0.7	1	8	9	24	0.42	-	-	-	-	-	-	-	-	-	-
**Cheng.J.R**	-	-	-	-	-	-	-	-	-	-	-	-	-	-	-	-	-	-	-	-	12	13	33	7	0.005
**Cortes-Dericks.L**	-	-	-	-	-	-	-	-	-	-	-	-	-	-	-	-	-	-	-	-	-	-	-	-	-
**Gao.Y**	9	14	21	28	0.3	10	12	22	18	0.6	-	-	-	-	-	8	7	24	23	1	11	18	21	12	0.043
**Gottschling.S**	-	-	-	-	-	-	-	-	-	-	-	-	-	-	-	-	-	-	-	-	-	-	-	-	-
**Gu.Y.P**	14	4	16	10	0.419	9	8	21	6	0.085	16	10	14	4	0.419	28	13	2	1	1	9	3	21	11	0.817
**Herpel.E**	-	-	-	-	-	3	22	10	51	0.853	-	-	-	-	-	13	72	1	2	0.971	10	51	3	22	0.853
**Huang.M.J**	47	41	78	73	0.793	32	27	93	87	0.732	-	-	-	-	-	-	-	-	-	-	-	-	-	-	-
**Janikova.M**	-	-	-	-	-	-	-	-	-	-	-	-	-	-	-	-	-	-	-	-	-	-	-	-	-
**Le.H.B**	-	-	-	-	-	-	-	-	-	-	-	-	-	-	-	-	-	-	-	-	-	-	-	-	-
**Li.F**	-	-	-	-	-	9	25	37	74	0.452	18	31	28	68	0.354	-	-	-	-	-	-	-	-	-	-
**Li.H**	19	25	25	21	0.289	7	12	37	34	0.237	-	-	-	-	-	-	-	-	-	-	4	15	40	31	0.013
**Li.L.D**	48	9	48	7	0.643	16	4	80	14	0.817	-	-	-	-	-	-	-	-	-	-	-	-	-	-	-
**Lin.X.Y**	-	-	-	-	-	-	-	-	-	-	-	-	-	-	-						7	17	20	10	0.006
**Mizugaki.H**	60	20	64	17	0.545	43	9	81	28	0.237	38	12	72	24	0.894	33	10	91	27	0.96	77	29	47	8	0.067
**Okudela.K**	-	-	-	-	-	-	-	-	-	-	-	-	-	-	-	-	-	-	-	-	-	-	-	-	-
**Pirozzi.G**	-	-	-	-	-	-	-	-	-	-	-	-	-	-	-	-	-	-	-	-	-	-	-	-	-
**Qiu.Z.X**	46	45	53	31	0.094	23	22	76	54	0.391	-	-	-	-	-	63	51	36	25	0.633	53	43	46	33	0.688
**Salnikov.A.V**	-	-	-	-	-	6	3	50	29	1	-	-	-	-	-	16	12	40	20	0.387	-	-	-	-	-
**Shien.K**	12	9	3	6	0.426	2	7	7	14	0.862	1	7	8	14	0.417	-	-	-	-	-	-	-	-	-	-
**Song.S.M**	-	-	-	-	-	-	-	-	-	-	-	-	-	-	-	-	-	-	-	-	16	21	39	14	0.004
**Sowa**	-	-	-	-	-	-	-	-	-	-	-	-	-	-	-	-	-	-	-	-	-	-	-	-	-
**Su.C.X**	36	43	41	39	0.474	37	35	40	47	0.497	44	47	33	35	0.982	-	-	-	-	-	-	-	-	-	-
**Sullivan.J.P**	-	-	-	-	-	-	-	-	-	-	-	-	-	-	-	-	-	-	-	-	-	-	-	-	-
**Sun.H.Y**	20	9	22	16	0.353	7	7	36	17	0.213	14	12	28	13	0.233	-	-	-	-	-	-	-	-	-	-
**Tirino.V**	22	11	42	14	0.398	25	5	39	20	0.087						51	20	13	5	0.974	55	20	9	5	0.489
**Wang.S.G**	21	8	13	3	0.766	-	-	-	-	-	34	4	34	11	0.175	-	-	-	-	-	11	10	57	5	0.0001
**Wei.Y.P**	-	-	-	-	-	-	-	-	-	-	-	-	-	-	-	-	-	-	-	-	15	23	25	15	0.042
**Wu.S.W**	64	76	85	79	0.2888	33	39	116	117	0.558	-	-	-	-	-	-	-	-	-	-	35	85	114	71	0.0001
**Xu.Y.H**	35	35	16	16	1	181	18	33	33	1	29	25	22	26	0.427	16	15	35	36	0.83	14	20	37	31	0.208
**Yao.J**	-	-	-	-	-	-	-	-	-	-	-	-	-	-	-	-	-	-	-	-	7	8	24	3	0.009

Study	Metastasis(Y/N)					TNM stage(III/IV vs. I/II)					Differentiation (moderate and poor vs. well)					histology(ADC/SSC)				
**CD133**	−		+		*p*	−		+		*p*	−		+		*p*	−		+		*p*
**Alamgeer.M**	-	-	-	-	-	81	63	23	28	0.022	-	-	-	-	-	31	53	73	48	0.001
**Bertolini.G**	-	-	-	-	-	23	20	9	6	0.767	-	-	-	-	-	1	13	8	17	0.119
**Cheng.J.R**	-	-	-	-	-	30	13	15	7	1	17	6	28	14	0.588	21	13	10	4	0.741
**Cortes-Dericks.L**	-	-	-	-	-	-	-	-	-	-	-	-	-	-	-	-	-	-	-	-
**Gao.Y**	16	20	16	10	0.184	-	-	-	-	-	-	-	-	-	-	15	12	11	14	0.579
**Gottschling.S**	-	-	-	-	-	-	-	-	-	-	-	-	-	-	-	-	-	-	-	-
**Gu.Y.P**	24	13	6	1	0.52	-	-	-	-	-	25	13	5	1	0.7	15	4	8	4	0.734
**Herpel.E**	-	-	-	-	-	-	-	-	-	-	-	-	-	-	-	4	30	8	28	0.399
**Huang.M.J**	-	-	-	-	-	91	91	34	23	0.203	2	14	123	100	0.002	63	62	23	23	1.935
**Janikova.M**	-	-	-	-	-	-	-	-	-	-	-	-	-	-	-	-	-	-	-	-
**Le.H.B**	-	-	-	-	-	-	-	-	-	-	-	-	-	-	-	-	-	-	-	-
**Li.F**	-	-	-	-	-	14	31	32	68	0.915	-	-	-	-	-	28	57	18	42	0.708
**Li.H**	-	-	-	-	-	18	26	26	20	0.139	-	-	-	-	-	-	-	-	-	-
**Li.L.D**	-	-	-	-	-	11	2	74	13	1	21	7	48	7	0.158	43	13	16	1	0.216
**Lin.X.Y**	-	-	-	-	-	-	-	-	-	-	6	15	21	12	0.012	-	-	-	-	-
**Mizugaki.H**	-	-	-	-	-	67	27	57	10	0.04	33	12	85	18	0.201	51	15	66	19	0.956
**Okudela.K**	-	-	-	-	-	-	-	-	-	-	-	-	-	-	-	-	-	-	-	-
**Pirozzi.G**	-	-	-	-	-	-	-	-	-	-	-	-	-	-	-	-	-	-	-	-
**Qiu.Z.X**	103	72	4	4	0.896	40	64	35	36	0.109	39	33	59	43	0.63	45	29	46	45	0.187
**Salnikov.A.V**	22	8	34	24	0.174	13	11	43	21	0.258	-	-	-	-	-	27	16	19	13	0.764
**Shien.K**	-	-	-	-	-	9	12	1	8	0.205	-	-	-	-	-	3	7	6	14	1
**Song.S.M**	-	-	-	-	-	49	32	6	3	1	8	10	47	25	0.105	25	16	30	19	0.981
**Sowa**	-	-	-	-	-	-	-	-	-	-	-	-	-	-	-	-	-	-	-	-
**Su.C.X**	-	-	-	-	-	-	-	-	-	-	-	-	-	-	-	26	39	51	43	0.077
**Sullivan.J.P**	-	-	-	-	-	-	-	-	-	-	-	-	-	-	-	-	-	-	-	-
**Sun.H.Y**	17	18	25	7	0.012	27	19	15	6	0.317	7	9	35	16	0.073	16	11	26	14	0.643
**Tirino.V**	-	-	-	-	-	-	-	-	-	-	-	-	-	-	-	23	9	35	14	0.965
**Wang.S.G**	-	-	-	-	-	-	-	-	-	-	61	11	7	4	0.203	-	-	-	-	-
**Wei.Y.P**	-	-	-	-	-	-	-	-	-	-	16	19	24	18	0.318	14	11	20	20	0.638
**Wu.S.W**	-	-	-	-	-	29	111	120	45	0.001	4	30	145	126	0.0001	99	111	50	45	0.375
**Xu.Y.H**	48	47	3	4	1	-	-	-	-	-	30	42	21	9	0.009	24	27	25	21	0.473
**Yao.J**	-	-	-	-	-	-	-	-	-	-	12	4	19	7	1	8	7	16	2	0.047

**Figure 5:**
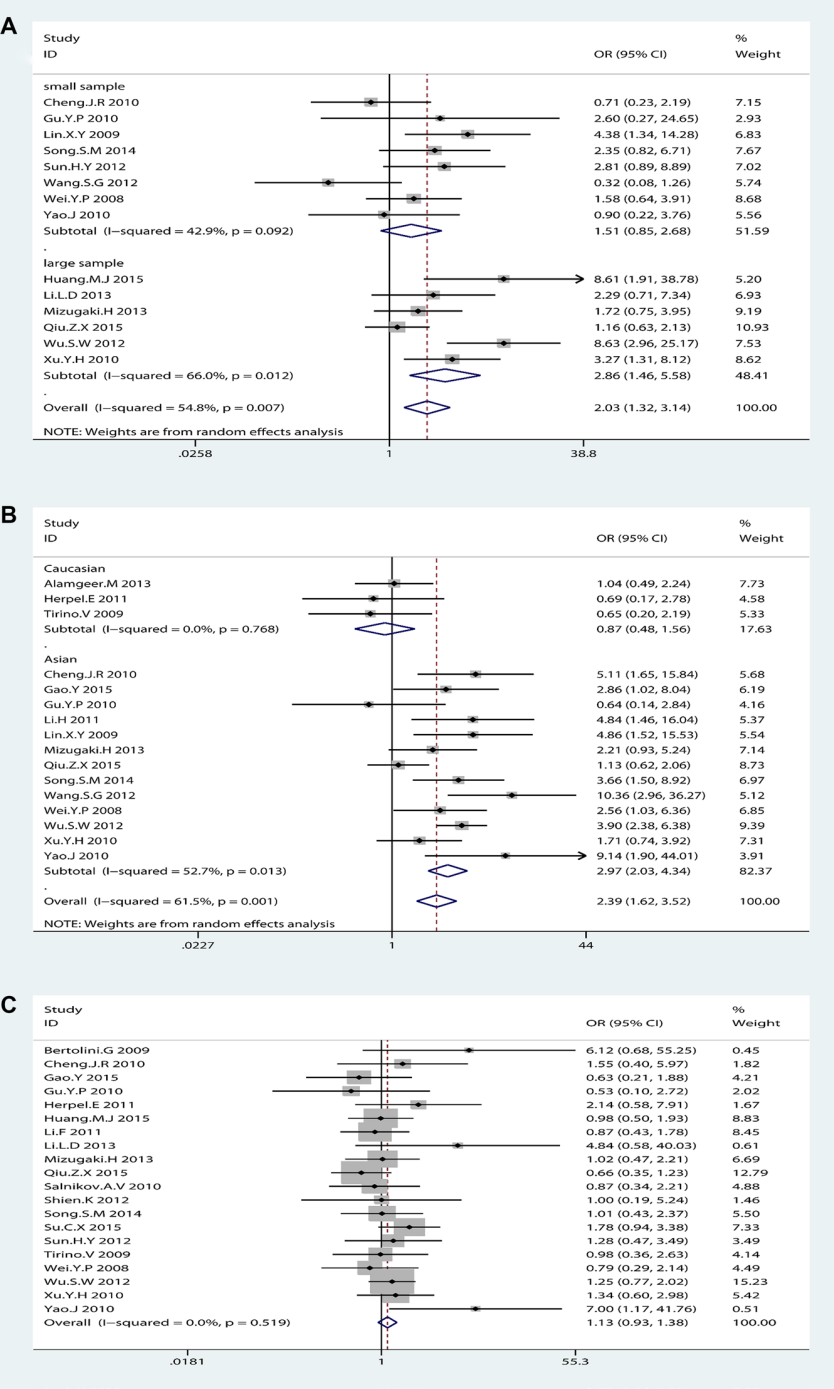
The subgroup analysis exploring the significant heterogeneity of CD133 expression with (A) differentiation by sample size (B) lymph node metastasis by race (C) histological type (adenocarcinoma vs. Squamous-cell carcinoma) after removed one study

We performed subgroup analysis regularly. Concerning differentiation, subgroup analysis by sample size but not race was performed due to all eligible studies were Asian. Large sample size group (*n* > 100) was contributed to the main heterogeneity (I^2^ = 66.0%, *p* = 0.012) with significant association (OR = 2.86, 95% CI: 1.46–5.58, *p* = 0.002) but not small sample size group (OR = 1.51, 95% CI: 0.85–2.68, *p* = 0.162; I^2^ = 42.9%, *p* = 0.092) (Figure 5A). As for lymph node metastasis, subgroup analysis by sample size could not explain the source of heterogeneity but race could (see Supplementary Figure S12 in Supplementary Material). The subgroup analysis by race showed that Asian group was contributed to a large proportion of heterogeneity (I^2^ = 52.7%, *p* = 0.013) with significant association (OR = 2.97, 95% CI: 2.03–4.34, *p* < 0.001) compared with Caucasian group (OR = 0.87, 95% CI: 0.48–1.56, *p* = 0.638; I^2^ = 0%, *p* = 0.768) (Figure 5B). These results suggested that NSCLC patients with higher CD133 expression had poor prognosis only in Asian patients but not in Caucasian patients.

It seemed that the expression of CD133 in lung adenocarcinoma patients (ADC) was more than in lung squamous-cell carcinoma (SCC) patients, which was in agreement with Wang. W et al. [25]. However, sensitive analysis showed that the OR of the association between CD133 expression and histological type (ADC vs. SCC) in NSCLC patients was dramatically changed after removed one study (Alamgeer.M 2013) (OR = 1.13, 95% CI: 0.93–1.38, *p* = 0.3; I^2^ = 0%, *p* = 0.522) (Figure 5C). Thus, it could not come to a conclusion that there was significant difference of CD133 expression level between ADC and SCC in NSCLC patients, which was different from Wang. W et al. [25].

### Sensitive analysis and publication bias

**Figure 6:**
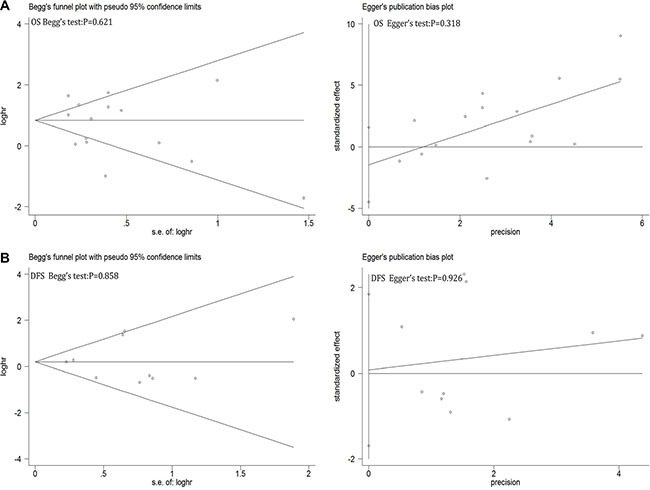
Begg's funnel plot and Egger's test to evaluate the publication bias for (A) OS and (B) DFS

Sensitive analysis showed that regardless of which one study removed, pooled HRs of left studies on OS and DFS were remain robust and stable (see Supplementary Figure S13 in Supplementary Material). Begg's funnel plot and Egger's publication bias plot were used to evaluate to the publication bias on OS (Figure 6A) and DFS (Figure 6B), respectively. No publication bias evidence was found in OS (Begg's test: *p* = 0.621; Egger's test: *p* = 0.318) or DFS (Begg's test: *p* = 0.858; Egger's test: *p* = 0.926). Same as in clinicopathological features (see Supplementary Table S2 in Supplementary Material).

## DISSCUSION

Mainly benefit from tobacco control and improvements in early detection and treatment, mortality rates decreased for lung cancer by 45% and 8% since from 1990 to 2015 in men and women, respectively [57]. However, only a small proportion of lung cancers are currently detected early [57], and more effective methods are needed to reduce the morbidity and mortality of lung cancer.

The CSCs hypothesis elucidates that a small proportion of tumor cells drive the cancer growth, progression and recurrence [58], which is different from the classical stochastic hypothesis [59]. In a landmark experiment, Singh SK and his colleagues showed that injection of as few as 100 CD133^+^ tumor cells were tumorigenic but injection of 10^5^ CD133^−^ tumor cells were not, giving stable foundation for CSCs hypothesis in many solid tumors [60]. Recent studies showed that CD133 was a biomarker of putative CSCs in many solid tumors from brain [60] [61], lung [62, 63], liver [64], pancreas [65] [66] and colon [67–70]. However, controversies remain exist when referring to the prognostic value of CD133 in solid tumors [9, 20–48, 54, 55, 71].

In this meta-analysis, we tried to elucidate the potential prognostic and clinical value of CD133 by systematically reviewing and analyzing 32 eligible literature. Interestingly and notably particularly, we found that NSCLC patients with higher CD133 expression have shorter overall survival time only in Asian patients but not in Caucasian patients. It remains unknown why racial difference causes this significant difference. Recent studies showed that EGFR and EGFRvIII signaling are concerned with maintaining a CSCs phenotype [72]. The EGFR positive CSCs represented enhanced tumorigenic potential and highly invasive behavior whereas EGFR negative CSCs reduced their tumorigenic ability [73]. Furthermore, Mitsudomi et al. reported that the EGFR mutation rate was 32% in patients of East Asian compared with 7% in patients of non-Asian [74]. Probably as a consequence, we speculated that difference of mEGFR of CD133^+^ CSCs in different racial NSCLC patients might be the potential mechanism causing the significant difference on OS. Here to yonder, we speculated that only the intrinsic EGFR gene status of CSCs could predict the efficacy of epidermal growth factor receptor tyrosine kinase inhibitors (EGFR-TKIs) in NSCLC patients, which are effective target drugs for NSCLC patients with EGFR mutations. But so far, general method for detecting EGFR mutations in lung cancer is direct sequencing with a low sensitivity, which could not uncover the EGFR gene status of tumor factually [75]. Therefore, detecting EGFR gene status after identification and isolation of CSCs using CD133 in NSCLC patients might be preferable strategy for choosing EGFR-TKIs.

Certain limitations in our study might influence the results. Firstly, these eligible studies were incorporated with varying TNM stage. Secondly, detection methods and threshold value of CD133 expression level were not consistent. Thirdly, though we performed subgroup analysis to explore the significant heterogeneity and further stabilized and consolidated our results that NSCLC patients with higher CD133 expression had poor overall survival time only in Asian patients but not in Caucasian patients, we could not explain fully the potential heterogeneity on differentiated degree and lymph node metastasis. Fourthly, relevant data in several eligible studies were too limited to pool all studies for evaluating the association between CD133 expression level and these parameters, which might overrate the clinical value of CD133.

Therefore, added large-scale sample, high-quality, and interethnic studies will be required to confirm the prognostic and clinical value of CD133. Far more than, the association between CD133^+^ CSCs and EGFR mutation in NSCLC patients is further deserving of attention and exploration, which may provide a new therapeutic perspective on the treatment of NSCLC patients according to the expression of CD133 and the intrinsic EGFR gene status of CD133^+^ CSCs.

## MATERIALS AND METHODS

### Search strategy

We searched PubMed, Embase, and Web of science to confirm relevant studies on CD133 expression level in NSCLC patients from each database since its inception up to May 4, 2016 without language restriction by using the keywords of CD133 and lung cancer (detail search strategy see Supplementary Material).

### Inclusion and exclusion criteria

A study was selected when met the following criteria: (1) the study population were mainly NSCLC patients; (2) it investigated the prognostic role of CD133 with the survival outcomes and/or clinicopathological characteristics in NSCLC patients. The exclusion criteria: (1) meeting report, review, comment, or letter; (2) it was a reduplicative study whose data had been published in another study, and then left the complete one in this meta-analysis. Independently evaluations were performed by two authors (Engeng Chen and Zhiru Zeng) according to the inclusion and exclusion criteria.

### Data extraction

A double abstraction process was performed for data extraction (Engeng Chen and Zhiru Zeng). Disagreements were resolved by consulting the third author (Bingjun Bai). The following data were collected from eligible studies: the first author, publication year, race, number of NSCLC patients with CD133 measured, gender distribution, age, TNM stage, CD133 positive threshold, CD133 positive ratio, experimental method, primary outcomes (reported HR with its 95% CI on OS and DFS), and essential clinicopathological characteristics (T stage, N stage, M stage, TNM stage, smoking history, differentiation grade, and histological type).

### Quality assessment of eligible studies

The Newcastle-Ottawa Quality Assessment Scale (NOS) [56] was used to evaluate the quality of each eligible study by two authors (Engeng Chen and Zhiru Zeng) independently. This scale ranges from 0 to 9 score, and we consider the study as a high quality study if the score is not less than 6.

### Statistical analysis

The main purpose of this meta-analysis was to estimate the pooled HRs of OS and DFS, then to validate the hypotheses: that NSCLC patients with higher CD133 expression would have a shorter OS and DFS time. The secondary purpose was to estimate the pooled ORs to analyze the correlation between CD133 expression level and clinicopathological features, with the doubts: that is there any cause-and-effect relationship between CD133 and these features.

We analyzed each eligible study to obtain HR and DFS with corresponding 95% CI from the results of multivariate Cox's proportional hazards regression model reported in the study. Also we reconstructed and calculated the data from Kaplan-Meier survival curve using Engauge-Digitizer version 7.2 if there was no direct data in the study [76]. The ORs with corresponding 95% CIs were calculated according to the relevant parameters using chi-square test by SPSS version 21 (SPSS Inc. Chicago, USA) in eligible studies.

The following analyses were performed using Stata version 12 software (Stata Corporation, College Station, Texas, USA). Pooled HRs of OS and DFS and pooled ORs for the relationship between CD133 and clinicopathological features were calculated by using fixed-effects model if I-square < 50%. Additionally, we used the Cochran's *Q*-test and I-square statistics to test for between-study heterogeneity [77–78]. Instead of fixed-effects, random-effects model was used if I-square > 50% or corresponding *p* value < 0.05. Furthermore, subgroup analysis and sensitive analysis were applied to assess the source of heterogeneity. The potential publication bias was tested by using Begg's test and Egger's test [79–80]. All statistics *p*-value < 0.05 at two-tailed was considered statistically significant.

## CONCLUSIONS

In summary, this meta-analysis showed that high expression level of CSCs marker CD133 was strongly in correlation with poor OS but not DFS in NSCLC patients. Subgroup analysis by race showed that NSCLC patients with higher CD133 expression had shorter overall survival time only in Asian patients but not in Caucasian patients, suggesting that differential prognostic value of CD133 expression in distinct ethnic group. Additionally, higher expression of CD133 was associated with poor differentiation and lymph node metastasis but there was no significant difference of CD133 expression between ADC and SCC in NSCLC patients. Therefore, added large-scale, prospective and clinical studies are required to further validate the prognostic and clinical value of CSCs marker CD133.

## SUPPLEMENTARY MATERIALS FIGURES AND TABLES


