# Supplementary material for: The prognostic value of CSCs biomarker CD133 in NSCLC: a meta-analysis
Source: Oncotarget. 2016 Jul 30;7(35):56526–39. doi: 10.18632/oncotarget.10964 (PMC5302932; doi:10.18632/oncotarget.10964)
Supplement: Supplementary file 1 [file oncotarget-07-56526-s001.pdf]

The prognostic value of CSCs biomarker CD133 in NSCLC: a meta-analysis

Supplementary Materials

The search strategy were “AC133 antigen” [MeSH] OR “CD133 antigen” OR “fudenine” OR “prominin-1” OR “prominin-like PROML1” OR “PROML1” OR “prominin” OR “AC141 antigen” OR “AC133-2 antigen” OR “PROM1 protein, human” OR “prominin 1 protein, human” OR “CD133 protein, human” OR “AC133 antigen, human” OR “AC133 protein, human” OR “CD133” AND “Lung Neoplasms” [MeSH] OR “Pulmonary Neoplasms” OR “Neoplasms, Lung” OR “Lung Neoplasm” OR “Neoplasm, Lung” OR “Neoplasms, Pulmonary” OR “Neoplasm, Pulmonary” OR “Pulmonary Neoplasm” OR “Lung Cancer” OR “Cancer, Lung” OR “Cancers, Lung” OR “Lung Cancers” OR “Pulmonary Cancer” OR “Cancer, Pulmonary” OR “Cancers, Pulmonary” OR “Pulmonary Cancers” OR “Cancer of the Lung” OR “Cancer of Lung”.

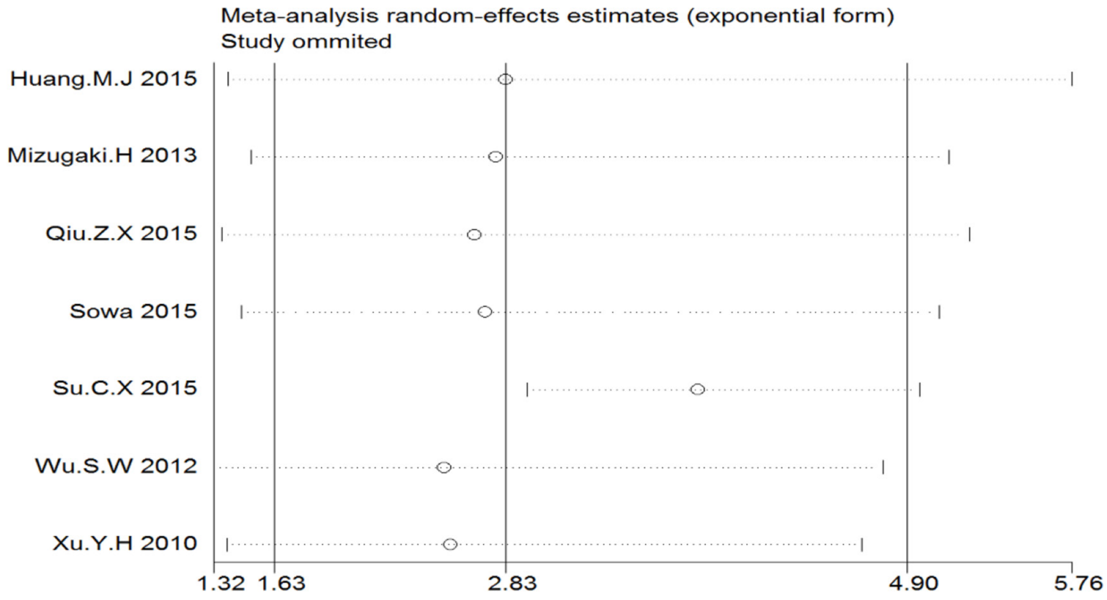

Supplementary Figure S1: Sensitive analysis in Asian studies with large sample size (*n* > 100) showed that whatever study was removed, the result of pooled HR of OS was stable.

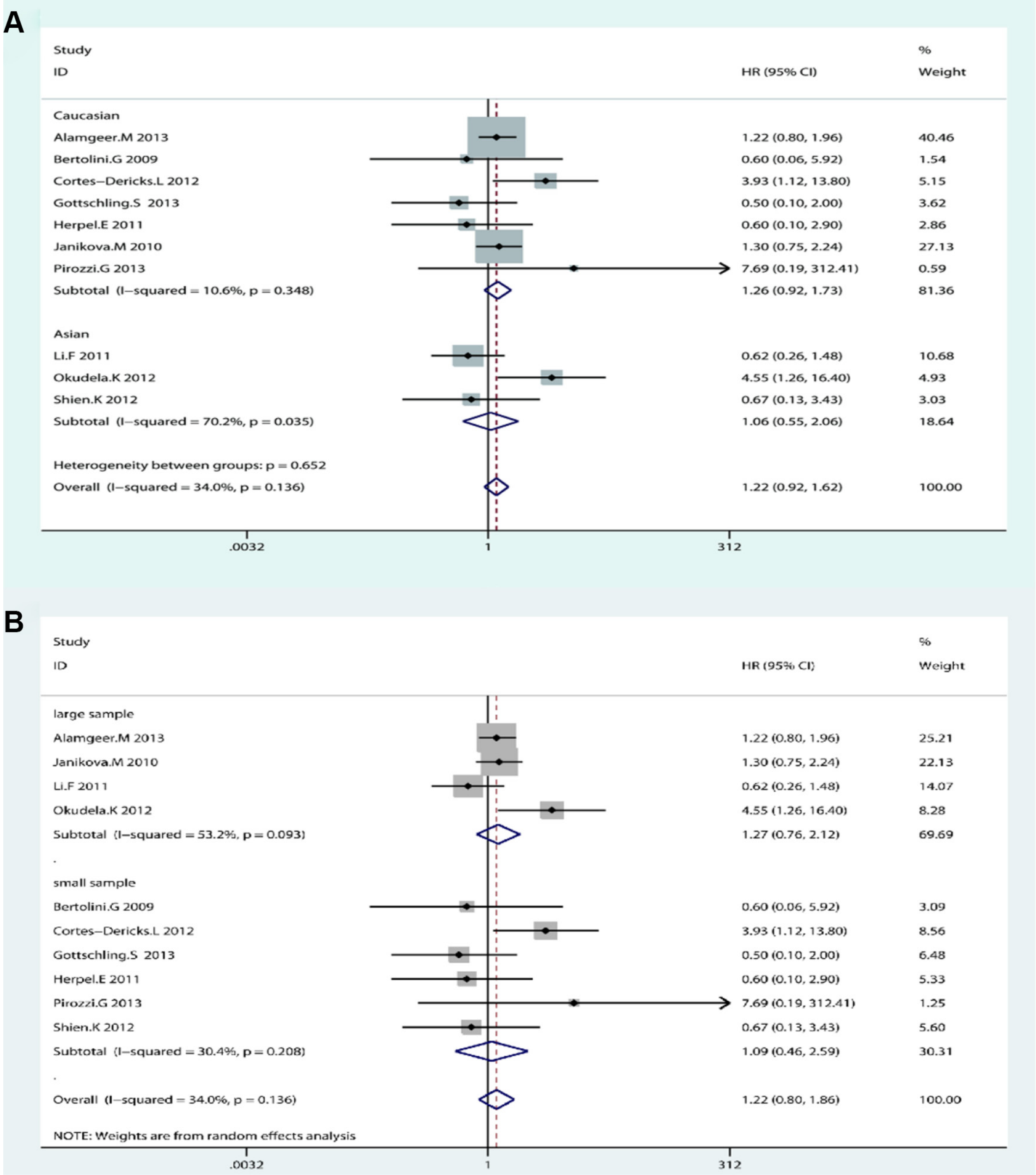

Supplementary Figure S2: The subgroup analysis exploring the significant heterogeneity of CD133 expression in NSCLC patients with (A) DFS by racial classification (B) DFS by sample size classification. It showed that there was no significant association between CD133 expression level and DFS in NSCLC patients by dividing race and sample into groups.

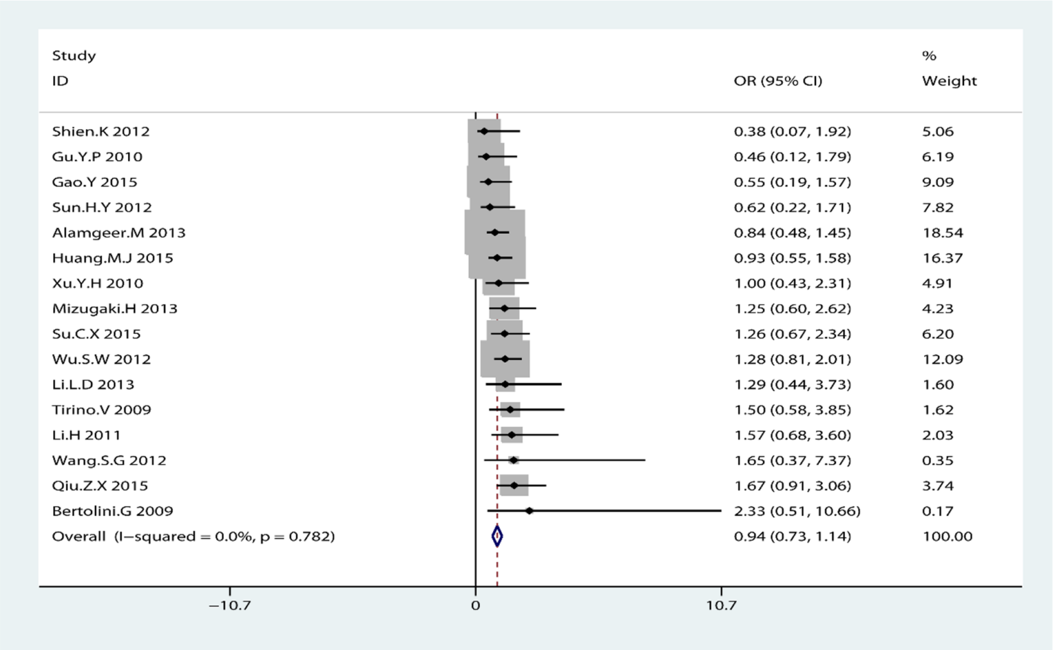

Supplementary Figure S3: Forest plot of odds ratios for the relationship of CD133 expression with age (old vs. young) in NSCLC patients.

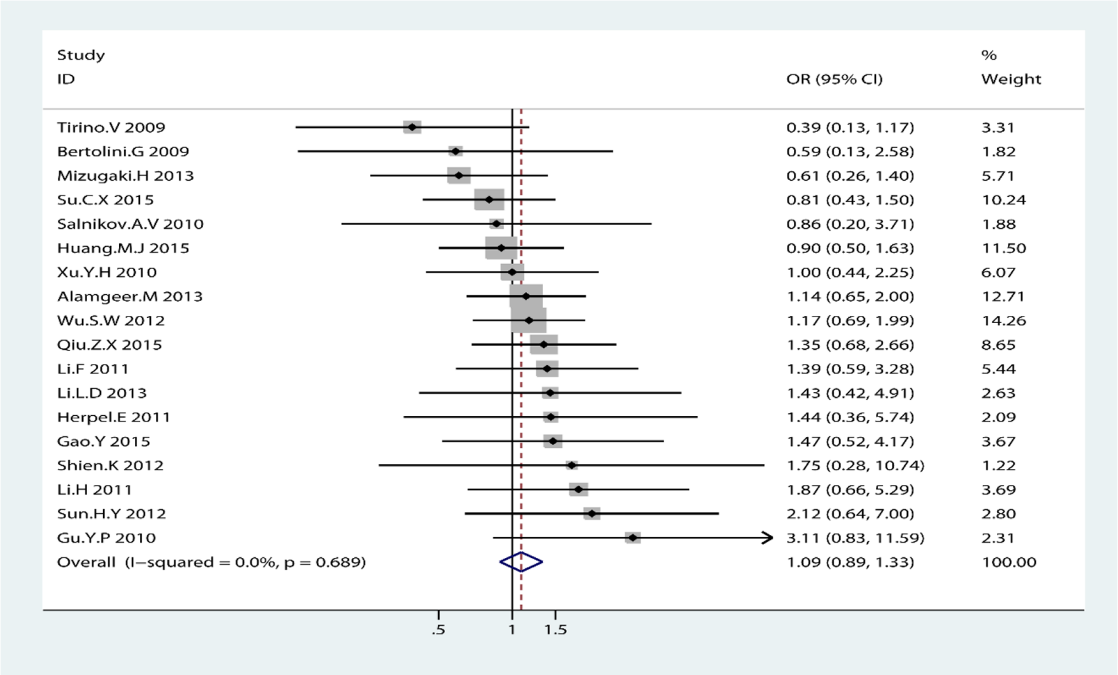

Supplementary Figure S4: Forest plot of odds ratios for the relationship of CD133 expression with gender (male vs. female) in NSCLC patients.

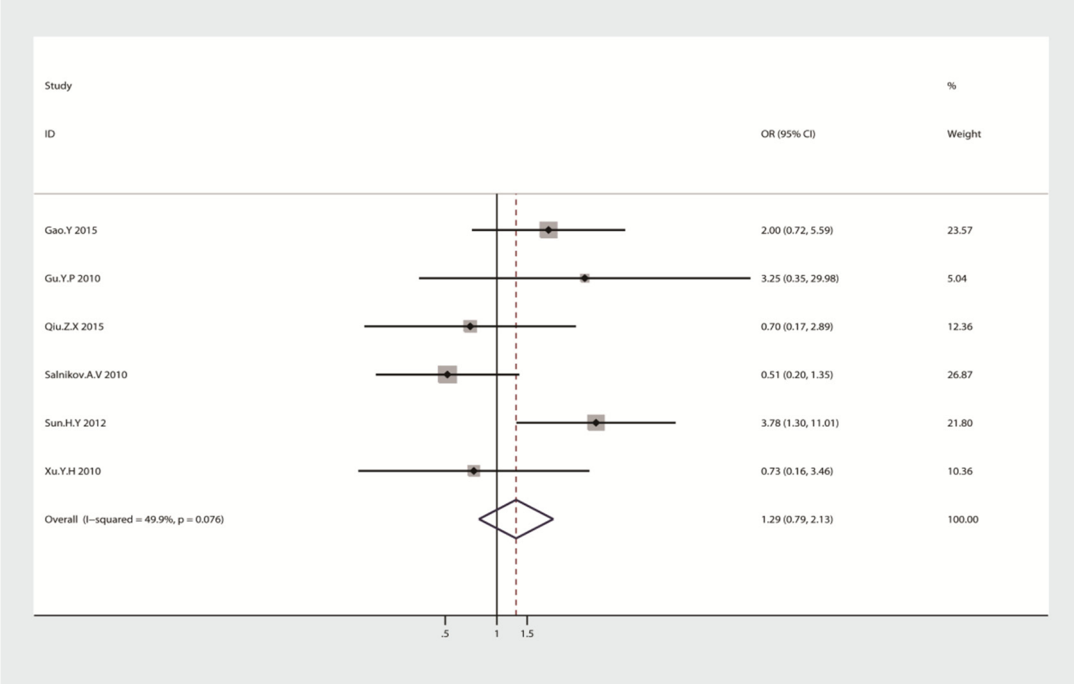

Supplementary Figure S5: Forest plot of odds ratios for the relationship of CD133 expression with metastasis (yes vs. no) in NSCLC patients.

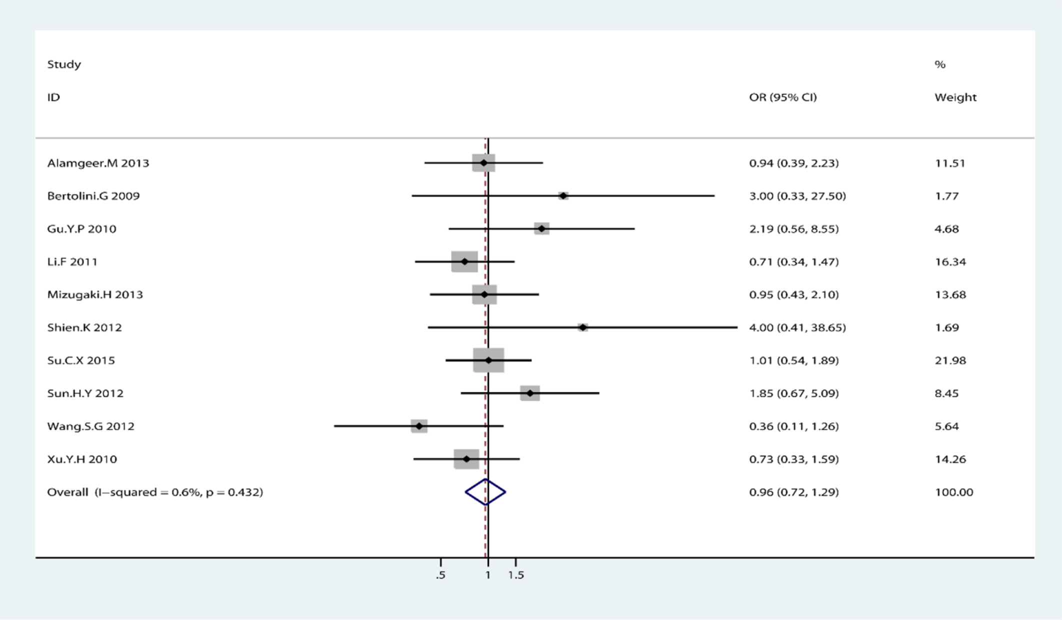

Supplementary Figure S6: Forest plot of odds ratios for the relationship of CD133 expression with smoking (yes vs. no) in NSCLC patients.

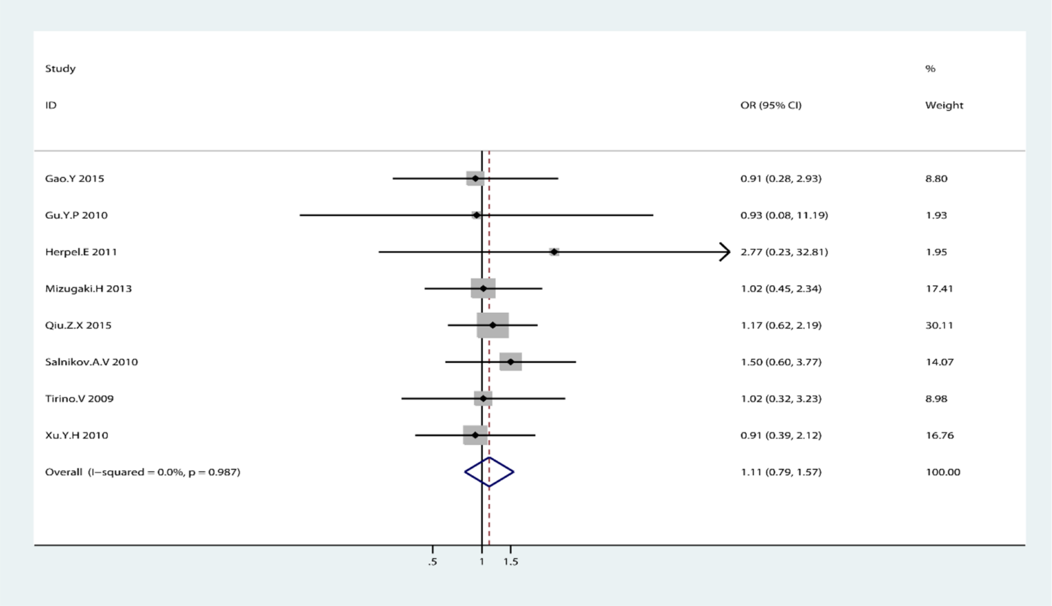

Supplementary Figure S7: Forest plot of odds ratios for the relationship of CD133 expression with T stage (T3/4 vs. T1/2) in NSCLC patients.

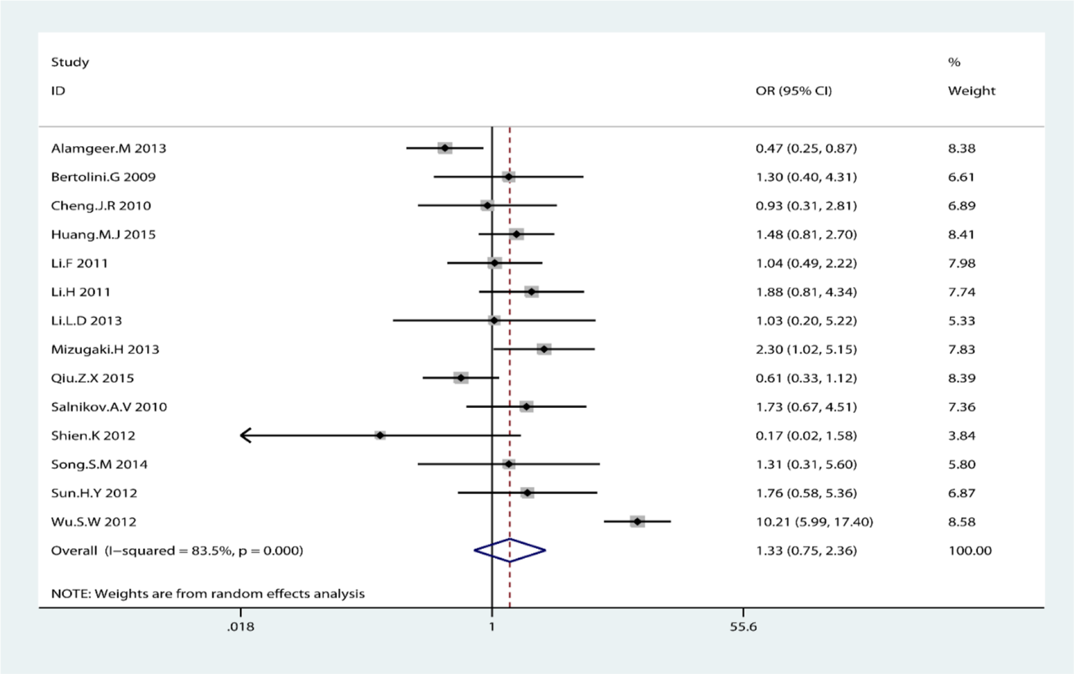

Supplementary Figure S8: Forest plot of odds ratios for the relationship of CD133 expression with TNM stage (III/IV vs. I/II) in NSCLC patients.

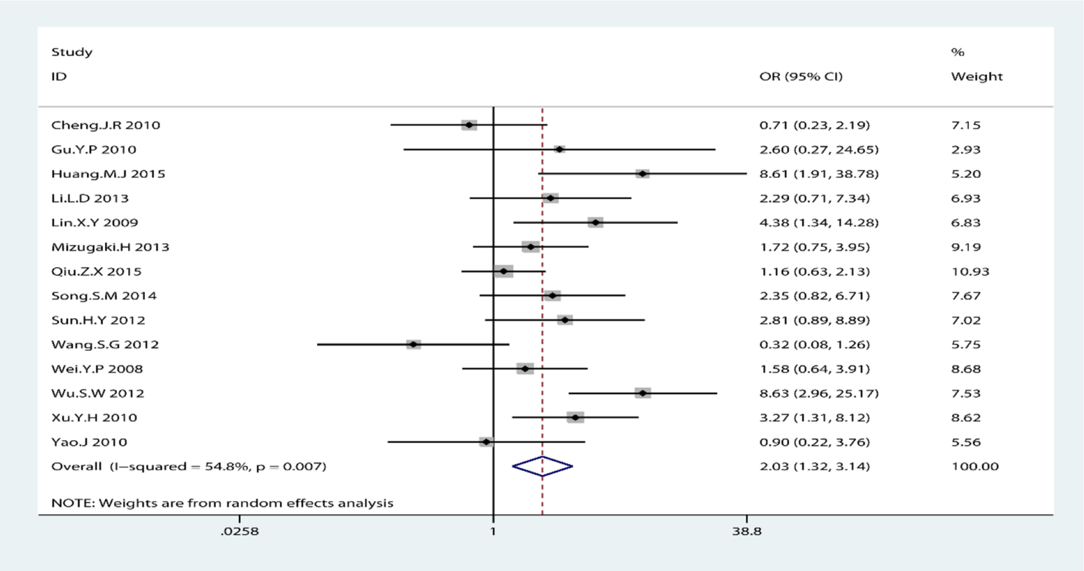

Supplementary Figure S9: Forest plot of OR for the association between CD133 expression and differentiation (poor/moderate vs. well) in NSCLC patients.

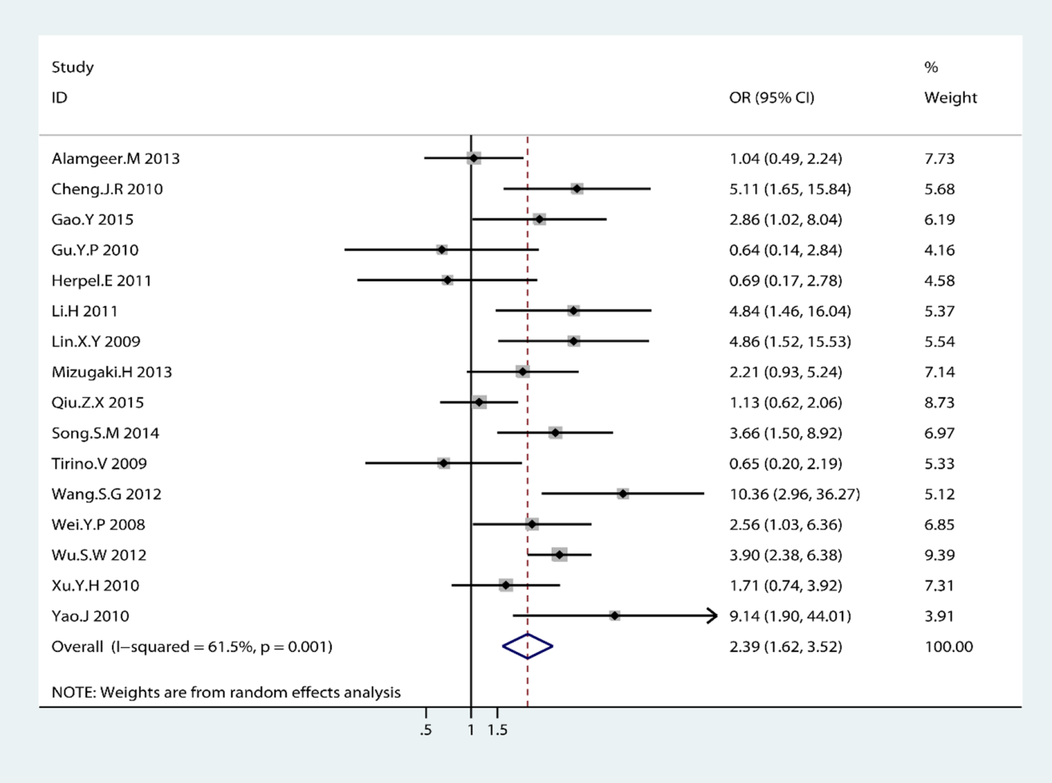

Supplementary Figure S10: Forest plot of OR for the association between CD133 expression and lymph node metastasis (yes vs. no) in NSCLC patients.

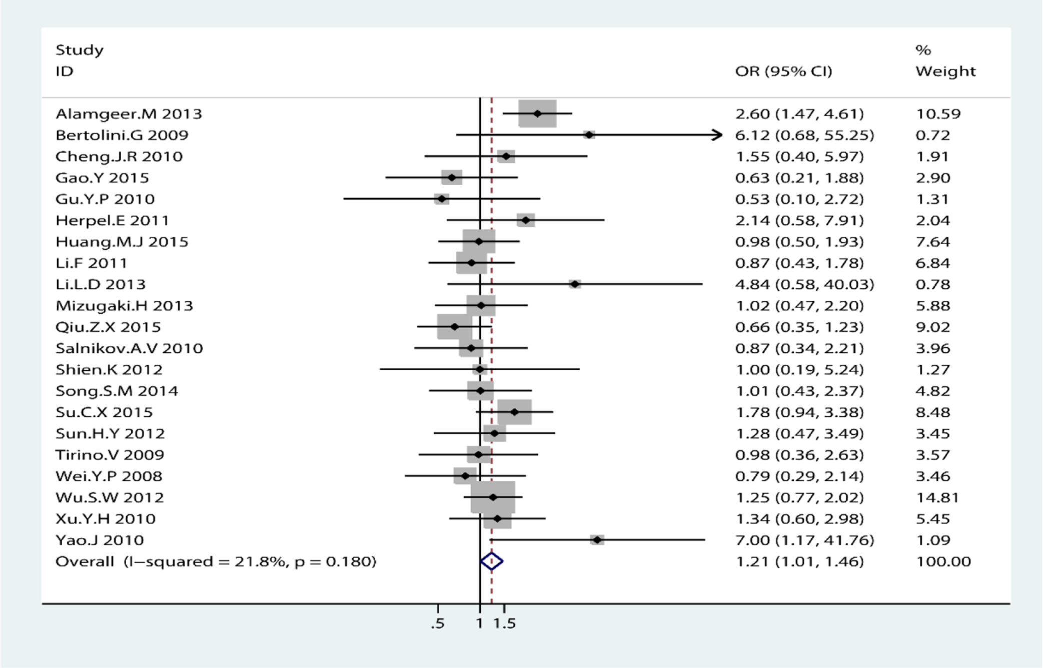

Supplementary Figure S11: Forest plot of OR for the association between CD133 expression and histological type (adenocarcinoma vs. squamous-cell carcinoma) in NSCLC patients.

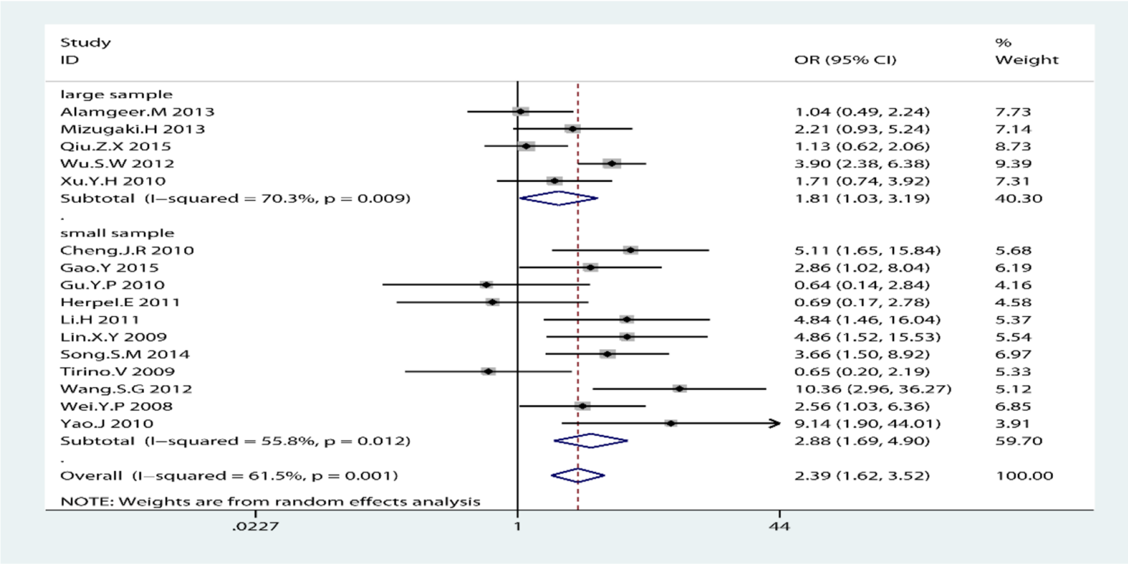

Supplementary Figure S12: The subgroup analysis exploring the significant heterogeneity of CD133 expression in NSCLC patients with lymph node metastasis by sample size.

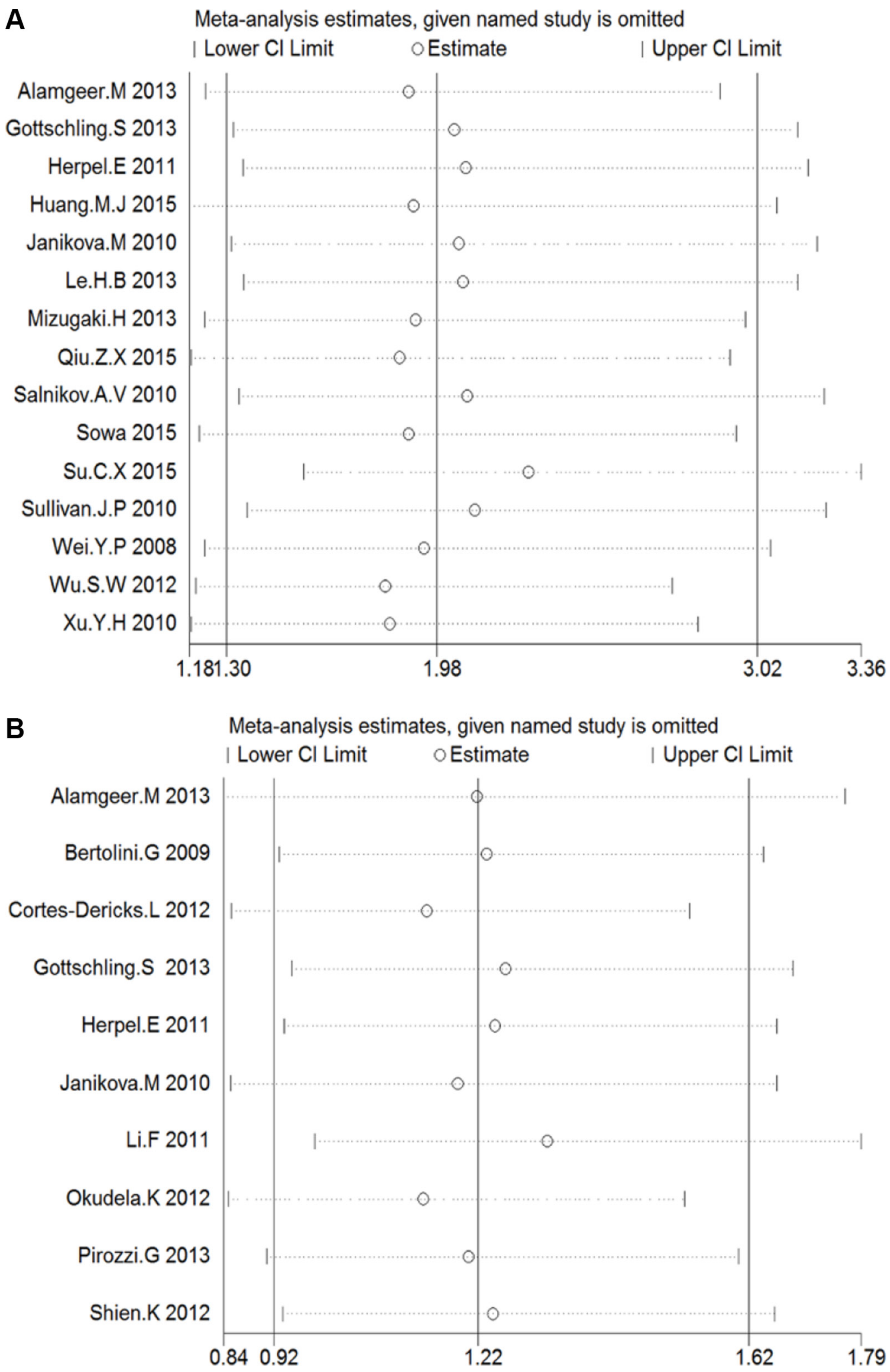

Supplementary Figure S13: Sensitive analysis for the association of CD133 expression in NSCLC patients with (A) OS and (B) DFS.

Supplementary Table S1: Newcastle-ottawa quality assessment scale for eligible studies in the meta-analysis

NEWCASTLE - OTTAWA QUALITY ASSESSMENT SCALE COHORT STUDIES									
	Selection				Comparability	Outcome			
Study	Representativeness of the exposed cohort	Selection of the non-exposed cohort	Ascertainment of exposure	Demonstration that outcome of interest was not present at start of study	Comparability of cohorts on the basis of the design or analysis	Assessment of outcome	Was follow -up long enough for outcomes to occur	Adequacy of follow up of cohorts	Total score
Alamgeer.M	1	1	1	0	2	1	1	1	8
Bertolini.G	1	1	1	0	1	1	1	1	7
Cheng.J.R	1	1	1	0	2	0	0	0	5
Cortes-Dericks.L	1	1	1	0	2	1	1	1	8
Gao.Y	1	1	1	0	2	0	0	0	5
Gottschling.S	1	1	1	0	2	1	1	1	8
Gu.Y.P	1	1	1	0	2	0	0	0	5
Herpel.E	1	1	1	0	2	1	1	1	8
Huang.M.J	1	1	1	0	2	0	0	0	5
Janikova.M	1	1	1	0	1	1	1	1	7
Le.H.B	1	1	1	0	1	1	1	1	7
Li.F	1	1	1	0	2	1	1	1	8
Li.H	1	1	1	0	2	0	0	0	5
Li.L.D	1	1	1	0	2	0	0	0	5
Lin.X.Y	1	1	1	0	1	0	0	0	4
Mizugaki.H	1	1	1	0	1	1	1	1	7
Okudela.K	1	1	1	0	2	1	1	1	8
Pirozzi.G	1	1	1	0	2	1	1	1	8
Qiu.Z.X	1	1	1	0	1	1	1	1	7
Salnikov.A.V	1	1	1	0	2	1	1	1	8
Shien.K	1	1	1	0	1	1	1	1	7
Song.S.M	1	1	1	0	2	0	0	0	5
Sowa	1	1	1	0	2	1	1	1	8
Su.C.X	1	1	1	0	2	1	1	1	8
Sullivan.J.P	1	1	1	0	1	1	1	1	7
Sun.H.Y	1	1	1	0	2	0	0	0	5
Tirino.V	1	1	1	0	2	0	0	0	5
Wang.S.G	1	1	1	0	1	0	0	0	4
Wei.Y.P	1	1	1	0	1	1	1	1	7
Wu.S.W	1	1	1	0	2	1	1	1	8
Xu.Y.H	1	1	1	0	2	1	1	1	8
Yao.J	1	1	1	0	2	0	0	0	5

Supplementary Table S2: Begg's test and Egger's test to evaluate the publication bias between CD133 expression level and clinicopathological features

publication bias analysis			
**CD133 with**	***p* for Begg's test**	***p* for Egger' s test**	**publication bias**
Age(old/young)	0.3	0.427	no
gender(male/female)	0.705	0.432	no
smoke(Y/N)	0.21	0.125	no
T stage(T3/4 vs.T1/2)	0.711	0.612	no
Lymph node Met (Y/N)	0.321	0.942	no
Metastasis(Y/N)	0.707	0.876	no
TNM stage(III/IV vs. I/II)	0.381	0.355	no
Differentiation (moderate and poor vs. well)	0.584	0.492	no
histology(ADC/SSC)	0.291	0.499	no
